# Annotations

**Published:** 1902-07-19

**Authors:** 


### Annotations.

### Malaria in the Roman Campagna.

From time to time we have heard a good deal about the attempts which have been made in various places to obtain protection from malaria by means of mechanical contrivances—wire screens, mosquito curtains, etc.—the object of which has been to prevent the access of the infected mosquitoes, and where Europeans live in the neighbourhood of large numbers of natives who are beyond our control this may be, and indeed is, the only effective means of keeping the disease at bay. The native children are a perennial source of infection to the mosquitoes, and if we are to escape the disease we must keep these infected mosquitoes away during their "biting" hours. Clearly, however, when one has to do with a whole population, there is a better way, for if over a considerable area we could but ensure by the careful administration of prophylactic drugs, such as quinine, that the people shall be immune to the parasite, even though only for a time, the very source of infection would be dried up and the disease would cease. This is the basis of the method of fighting malaria which has been recommended by Professor Koch. What is now being done in the Roman Campagna with the object of suppressing the malaria, which is so disastrous to the dwellers in that district, is founded on this idea. Professor Grassi determined to discard all mechanical arrangements and to trust to the prophylactic influence of quinine, either alone or in combination with other drugs. The results are stated to have been very encouraging. The people are regaining confidence; they stay in the place and keep their health; and although, as is the case wherever they exist, the anopheles continue to sting, they no longer infect, being themselves free from infection. The continued success of any such method will however depend to a large extent upon the care with which it is carried out, and more especially upon the care taken to exclude fresh infection, a thing which is difficult to do. As however the area dealt with in this manner extends, this difficulty will probably much diminish. We may be sure that every care will be taken, for there is no single question of more importance to Italy at the present day than the suppression of malaria.

### Medicine, Surgery and Gynæcology.

When the Obstetricians and Gynæcologists of the Empire recently dined together, with Sir John Williams in the chair, the presidents of the rival societies met together in amity, and each in turn responded to the toast of "British Obstetric Medicine." Not unnaturally their speeches ran on different lines, but on one point the remarks of both tended in the same direction, pointing, curiously enough, towards the possible extinction of the specialty for whose glorification they were met together. Dr. Peter Horrocks, president of the Obstetrical Society of London, said that the subject of obstetrics was as long as medicine, as wide as surgery, and as deep as science, for all problems in the pathology, etiology, and treatment of disease came before obstetric physicians. Sir Halliday Croom, president of the British Gynæcological Society, went further. He said that gynæcology had now left the shoals and quicksands of uncertain medicine, and had launched into the pure waters of surgery, adding that the day was perhaps not far distant when gynæcologists would disappear altogether, when gynæcology would become an integral part of general surgery, and gynæcologists would be absorbed in the ranks of general surgeons. We cannot but feel that this remark was just, prophetic, and peculiarly appropriate at a moment when there seems every probability of much of the less dignified portion of obstetrical work passing into the hands of the midwives. Every manipulation which is employed in the treatment of disease falls into the category of surgery. Thus when once the attendance on physiological accouchements is handed over to the nurse, it will be only as a surgeon that the obstetrician will be employed; while as for gynæcology, there has never been the slightest excuse for drawing any dividing line between it and pure medicine and surgery, except the fact that it has mostly been practised by those who used to be branded in the eyes of the College of Surgeons with the bar sinister of the accoucheur. The hospitals and the colleges clearly ought to accept the inevitable. The days of the physician accoucheur are past. Obstetrics should go to surgery, and the titles of hospital officials should be made to conform to the facts of the case.

### Fires in Hospitals.

In view of recent catastrophes, and more particularly of the burning of a sanatorium for inebriates a little while ago when many lives were lost, the City Building Department of Chicago has undertaken the inspection of the local hospitals, with results which indicate how desirable it would be for all hospital authorities to go carefully into this question of fire protection. Architects cling to the belief that the staircase is the only proper way out of a building; while the first thing that a conflagration does is to fill the staircase with smoke and render it useless as a way of escape. In nearly all buildings, such as hospitals and workhouses, erected of late years by public bodies controlled by the Local Government Board, some reasonable provision is made for escape in case of fire from every floor. These appliances may not always be ornamental, but there they are. In the erection of voluntary hospitals, however, architects have had a more or less free hand. Committees have deferred to them, and if the plans have been passed by the local authorities it has been taken for granted that all proper requirements have been complied with. The result has too often been that the fire escapes if not utterly neglected, have been kept in the background and made as little obtrusive as possible, with disastrous results so far as their efficiency is concerned. When one considers the difficulty there often is in moving sick people up and down stairs, even when the steps are wide and the gradients well-proportioned, it is clear as clear can be that the so-called fire exits from some hospital wards have been arranged more to soothe the consciences of committees than to serve a serious purpose as modes of escape for sick and helpless patients in case of fire. It is quite evident that in regard to all buildings of any height the question of fire should be far more carefully considered than has hitherto been the case, and that in providing the necessary means of escape no weight should be given to merely artistic considerations if they in the slightest degree run counter to utility.

